# Neocommissure plasty to treat aortic regurgitation due to commissural detachment in type 0 bicuspid aortic valve

**DOI:** 10.1016/j.xjtc.2026.102374

**Published:** 2026-03-27

**Authors:** Ryo Kawabata, Soichiro Henmi, Jun Karaki, Hironaga Shiraki, Shunya Chomei, Noriko Ohyama, Katsuhiro Yamanaka, Hiroaki Takahashi, Kenji Okada

**Affiliations:** Division of Cardiovascular Surgery, Department of Surgery, Kobe University Graduate School of Medicine, Kobe, Japan

**Figure:**
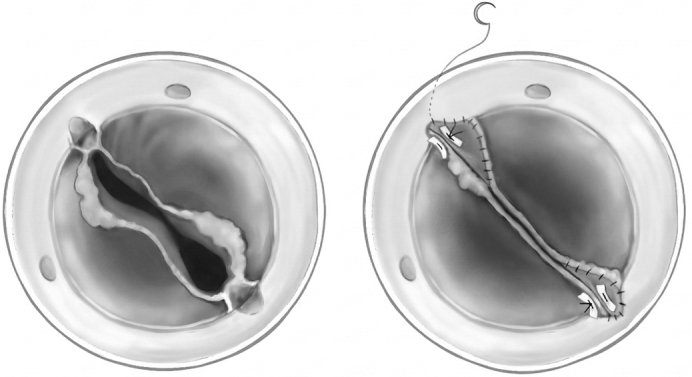
Pericardial patch neocommissure plasty for type 0 bicuspid valve with commissural detachment.

**Central Message**

Neocommissure plasty for type 0 bicuspid aortic valve commissural detachment enabled durable repair in three young patients, with mild or less aortic regurgitation and low gradients at 5 to 6 years postoperatively.

Bicuspid aortic valve (BAV) repair for aortic regurgitation (AR) has become increasingly standardized.^1^ Commissural detachment resulting in a focal “gap” at the commissure is uncommon, and reports on technique-focused repair are scarce.^2^^,^^3^ Here, we report neocommissure plasty using glutaraldehyde-treated autologous pericardium for the repair of type 0 BAV with commissural detachment. Institutional review board approval was waived. Written consent for publication was obtained.

## Representative Case (Patient 1)

**Figure 1:**
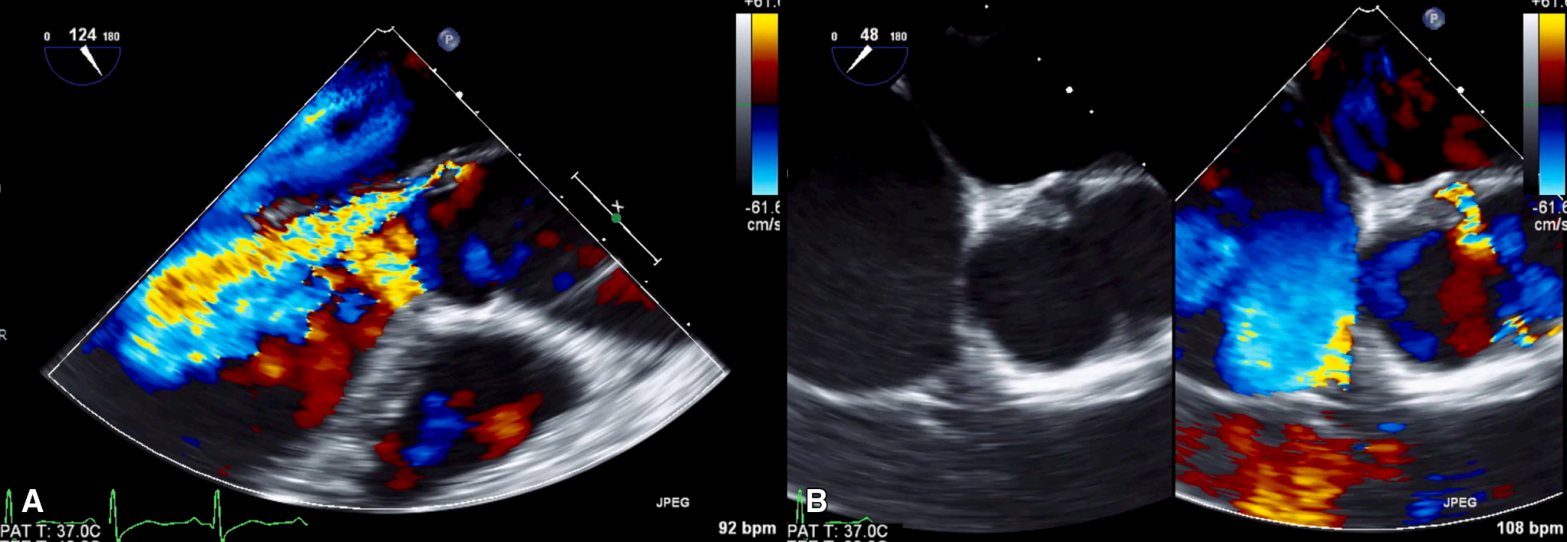
Preoperative transesophageal echocardiography. A, Long-axis color Doppler; B, short-axis color Doppler: paracommissural jets.

A 16-year-old male patient with no previous medical history was referred after a school screening murmur. He was asymptomatic but had severe AR. Preoperative transesophageal echocardiography revealed left ventricular (LV) dilatation (LV end-diastolic/end-systolic diameter, 55/39 mm) with preserved function (LV ejection fraction: 63%). Color Doppler imaging demonstrated regurgitant jets from both commissural regions, consistent with paracommissural gaps (Figure 1, *A* and *B*). The aortoventricular junction (AVJ), sinus of Valsalva, and sinotubular junction (STJ) diameters were 22, 26, and 24 mm, respectively. The ascending aorta was 40 mm in diameter.

## Surgical Technique

**Figure 2:**
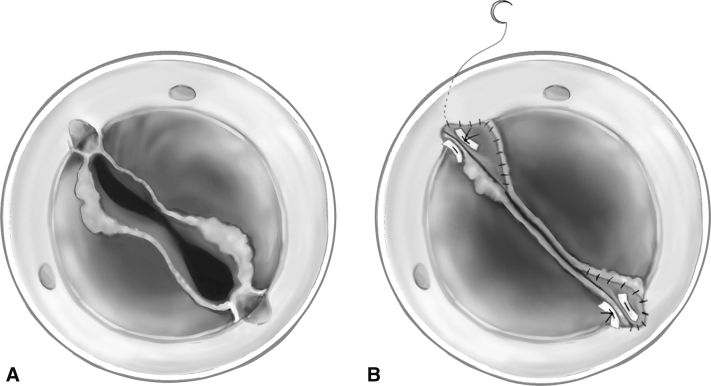
Schematic illustration of the valve repair. A, Before repair: bilateral commissural detachment. B, After repair: neocommissure plasty with glutaraldehyde-treated autologous pericardium.

The complete procedure is shown in Video 1. After we established cardiopulmonary bypass and cardioplegic arrest, the ascending aorta was opened, and the valve was identified as a Sievers type 0 BAV with bilateral partial commissural detachment and visible gaps (Figure 2, *A*). Autologous pericardium was harvested, treated with 0.6% glutaraldehyde, and trimmed into small patches.

**Figure 3:**
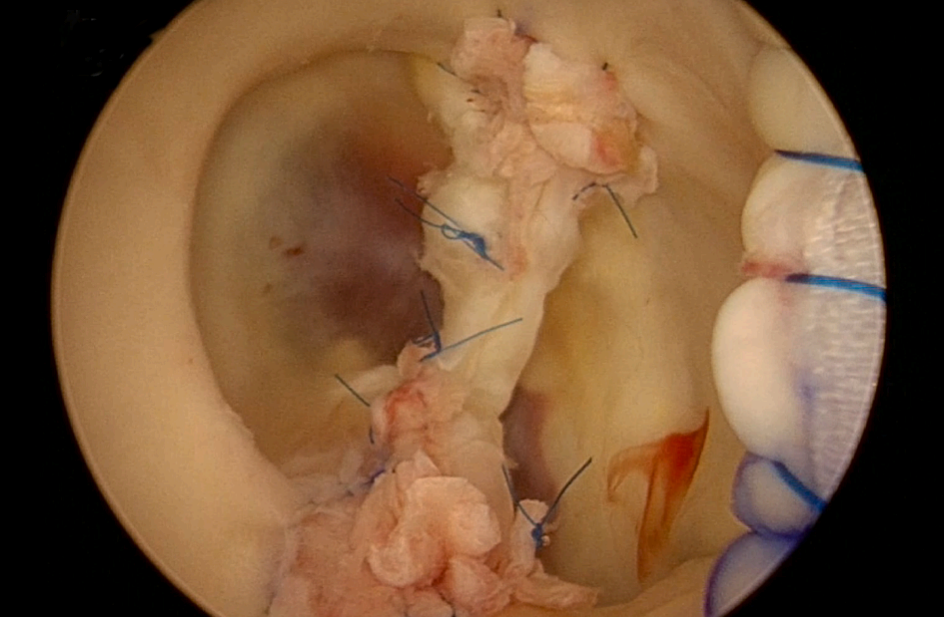
Postrepair endoscopy with root pressurization: good coaptation.

Using 6-0 polypropylene sutures, each patch was sutured to the adjacent cusp tissue along the commissural area to reconstruct a stable neocommissural attachment. The suture was then passed transaortically at the commissural height and tied externally over an autologous pericardial pledget. Any residual paracommissural gap was closed with an additional pledgeted 6-0 fixation stitch. The same steps were repeated for the opposite commissure (Figure 2, *B*). External plication with a narrow graft strip may be added for isolated AVJ and/or STJ dilatation, whereas root replacement can be considered when the aortic root diameter exceeds 45 mm. In this case, the patient had a bicuspid aortic valve with mild ascending aortic dilatation, and late STJ dilatation was a concern; therefore, concomitant ascending aortic replacement was performed using a 24-mm straight prosthetic graft (Gelweave Straight, Terumo). Valve competence was assessed by root pressurization and endoscopic inspection (Figure 3).

## Results

**Figure 4:**
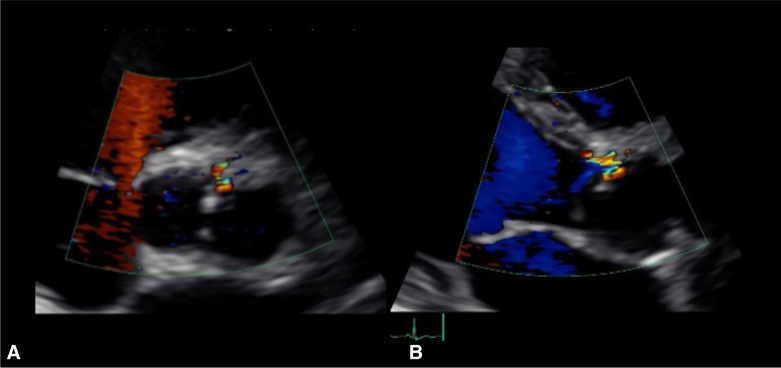
Latest transthoracic echocardiography at 6 years. Trace aortic regurgitation from the anterior commissure. A, Short-axis; B, long-axis.

Postoperative transesophageal echocardiography showed a good valve opening with trace AR. At 6 years, trace AR persisted, and the mean transvalvular gradient decreased (from 26 mm Hg at discharge to 18 mm Hg) without reintervention (Figure 4). This technique was applied in 3 young patients with Sievers type 0 BAV and commissural detachment; one required adjunctive AVJ/STJ plication. Over 5 to 6 years, AR remained trace-to-mild, and the mean gradient decreased from 19 ± 6 mm Hg at discharge to 14 ± 5 mm Hg at the latest follow-up (Table 1).

**Table 1:** Echocardiographic parameters at discharge and at the latest follow-up in 3 patients

Parameter	Patient 1		Patient 2		Patient 3	
	Discharge	Latest	Discharge	Latest	Discharge	Latest
Age, y/sex	16/M		22/M		39/M	
Follow-up duration, y	6		5.5		5.5	
AR grade	Trace	Trace	Mild	Mild	Mild	Mild
LVEDD/LVESD, mm	49/32	46/35	49/36	44/32	39/28	47/30
EF, %	60	57	57	56	69	63
Peak PG, mm Hg	48	30	30	15	24	27
Mean PG, mm Hg	26	18	16	8	15	16
EOA, cm^2^	1.17	1.14	2.2	1.9	1.6	1.95
EOAI, cm^2^/m^2^	0.75	0.68	1.5	1.3	0.85	0.95
AVJ/STJ plication	No		No		Yes	

*M*, Male; *AR*, aortic regurgitation; *LVEDD*, left ventricular end-diastolic diameter; *LVESD*, left ventricular end-systolic diameter; *EF*, ejection fraction; *PG*, pressure gradient; *EOA*, effective orifice area; *EOAI*, effective orifice area index; *AVJ*, aortoventricular junction; *STJ*, sinotubular junction.

## Discussion

AR caused by commissural detachment is rare, and published repair strategies are limited.^2^^,^^3^ Although the Ross procedure is an option, this simpler neocommissure plasty offers a reasonable alternative by avoiding “two-valve disease.”

We agree with the durability concerns regarding patch use noted in previous reports.^4^^,^^5^ However, rather than framing the issue as a binary debate over patch use, the focus should be on defining the appropriate anatomical and etiologic criteria for its use. In our small series, favorable midterm outcomes were achieved by strictly limiting the technique to (1) commissural detachment as the primary etiology of AR with minimal cusp degeneration, (2) Sievers type 0 anatomy, and (3) minimal use of patches.

Importantly, the primary etiology in our cohort differed fundamentally from that reported in previous studies. Given the limited sample size and the absence of long-term follow-up, caution is warranted when comparing our results with those from large-scale cohorts. Nevertheless, our findings suggest that patch-based repair may remain a viable and effective option for selected patients.

## Conflict of Interest Statement

The authors reported no conflicts of interest.

The *Journal* policy requires editors and reviewers to disclose conflicts of interest and to decline handling or reviewing manuscripts for which they may have a conflict of interest. The editors and reviewers of this article have no conflicts of interest.

## Supplementary Data

Video 1 Operative technique of neocommissure plasty to treat aortic regurgitation due to commissural detachment in type 0 bicuspid aortic valve. Video available at: https://www.jtcvs.org/article/S2666-2507(26)00181-1/fulltext.
